# Majorization-Minimization Method for Elliptic Localization in the Absence of Transmitter Position

**DOI:** 10.3390/s23010373

**Published:** 2022-12-29

**Authors:** Liehu Wu, Yanbin Zou

**Affiliations:** Department of Electronic and Information Engineering, Shantou University, Shantou 515063, China

**Keywords:** elliptic localization, maximum likelihood estimation (MLE), majorization–minimization (MM)

## Abstract

This paper investigates the problem of elliptic localization in the absence of transmitter position. An efficient iterative method is developed to jointly evaluate the target and transmitter positions. Using the measurement information from the indirect paths reflected from the target and the direct paths between the transmitter and receivers, a non-convex maximum likelihood estimation (MLE) problem is formulated. Owing to the non-convex nature of the issue, we apply the majorization–minimization (MM) principle to address the MLE problem, which iteratively minimizes a convex surrogate function instead of the original objective function. Moreover, the proposed MM method is further extended to tackle a general scenario where both multiple unknown transmitters and receiver position errors are considered. Finally, numerical simulations demonstrate that the proposed MM method outperforms the state-of-the-art methods.

## 1. Introduction

Target localization has been a prevalent research topic with numerous applications in wireless sensor network (WSN) [1,2,3], communications [4], radars [5,6,7], and many others. A straightforward method to locate a target is direct localization [8], in which the position is determined directly from the received signals. The direct approach bears expensive computational complexity due to the multidimensional grid search. Hence, various types of indirect approaches have been presented based on different types of measurement information. The typical measurement information includes time of arrival (TOA) [9,10], time-difference of arrival (TDOA) [11,12,13], received signal strength (RSS) [14], arrival of angle (AOA) [15], and their joint methods [16,17,18]. Among them, localization based on time information holds great research significance owing to its high-positioning accuracy. Elliptic localization is a time-based positioning model that is superior to some typical localization models, such as TOA-based and TDOA-based models [19]. In this model, the transmitter sends out a radar wave and several synchronous receivers collect the signal reflected from the interest target to determine its position. In addition, when the transmitter position is unknown, the signal received directly from the transmitter is also used to improve the positioning performance. Such a multistatic positioning approach [20] can offer more flexibility and better performance than the monostatic counterpart [21]. Hence, elliptic localization has been widely applied in multiple-input-multiple-output (MIMO) radars [22]. The flight time of the radar wave multiplied by its propagation speed produces the sum of the distances from the transmitter to the target and then to the receiver, which is referred to as the bistatic range [23,24]. The track of constant bistatic range in two-dimensional space is an ellipse with the transmitter and receiver positions as its foci, and the target position is on the ellipse. Thus, we can evaluate the target position by the intersection point of some ellipses produced by the bistatic ranges.

In reality, the transmitter position is potentially completely unavailable [20]. For instance, if the transmitter is set in some special inaccessible place or moves with time, its position cannot be accurately obtained. This also occurs in certain system designs such as the passive coherent location system [25,26,27], in which the transmitter position is purposely left unknown to simplify its structure and alleviate hardware requirements. Therefore, this situation bears important research significance. In [20], Zhang et al. first researched the elliptic localization problem in the absence of transmitter position. Using the measurements of the indirect and direct paths, a two-step weighted least squares (TSWLS) method was proposed for jointly estimating the target and transmitter positions. However, since the constrained relationships among those variables are difficult to directly and completely utilize, the TSWLS method has the *threshold effect* [28,29,30]. Zheng et al. [19] presented a semidefinite program (SDP) method to tackle the same problem, in which the semidefinite relaxation (SDR) technique is applied to convert this non-convex problem to a convex SDP problem. While the SDP method can approach the Cramér–Rao lower bound (CRLB) accuracy at a moderate noise level, this method requires a large number of complicated calculations arising from the CVX toolbox. Furthermore, there are several recursive methods that can be applied to solve the problem, such as the Gauss–Newton method [31] and the Quasi–Newton method [32,33].

This paper advances the topic by developing new solutions to the joint estimation problem. A maximum likelihood estimation (MLE) problem is formulated using the measurements of the indirect and direct paths. Since the problem is non-convex, its optimal solution cannot be solved directly. To tackle the difficulty, we propose an efficient iterative algorithm based on the majorization–minimization (MM) principle to address the formulated MLE problem. It requires constructing a convex surrogate function at each iteration that tightly upper bounds the original objective function, then minimizes the surrogate function to conduct the next iteration. Through the proposed algorithm, any given initial value will constantly approach the stationary point of the object function.

MM principle has been widely applied in signal processing, communications, and machine learning [34]. However, there is limited research about the MM principle for target localization. In fact, to the best of our knowledge, only Panwar et al. [35] has applied the MM principle to the multistatic target localization problem in terms of the precise known transmitter position. Furthermore, currently, no relevant works based on the MM principle have been discovered regarding the unknown transmitter position.

The main contributions of the current work are as follows:

- We formulate an MLE problem that jointly estimates the target and transmitter positions, and propose an effective iterative algorithm based on the MM principle to solve the optimization problem.
- We extend the proposed algorithm to a general scenario where both multiple transmitters at unknown positions and receiver position errors are considered.
- We theoretically analyze the computational complexity and convergence of the proposed algorithm.

The rest of this paper is organized as follows. Section 2 provides the measurement model for elliptic localization. In Section 3, we present a brief overview of the MM technique followed by the derivation of the proposed approach. It is also utilized to solve the scenario where both multiple transmitters and receiver position errors are considered. We then conclude the section with a discussion on the complexity and convergence of the proposed algorithm. In Section 4, numerical simulations are presented and conclusions are given in Section 5.

*Notations:* Bold upper-case and bold lower-case letters denote matrices and vectors, respectively. The notations $A^{T}$ and $A^{-1}$ represent the transpose and inverse of the matrix A, respectively. $a_{i}$, $a_{\left(i:j\right)}$, $A_{ij}$ and $A_{\left(i:j,k:l\right)}$ are the *i*-th element of a, the subvector containing the *i*-th to the *j*-th elements of a, the (i,j)-th element of A and the submatrix containing the elements from the *i*-th to the *j*-th row and the *k*-th to the *l*-th column of A, respectively. $I_{p\times p}$ is the p×p identity matrix. $O_{k\times l}$ is the k×l zero matrix. ⊗ and $∥\cdot ∥$ represent the Kronecker product and the Euclidean norm, respectively.

## 2. Measurement Model

**Figure 1:**
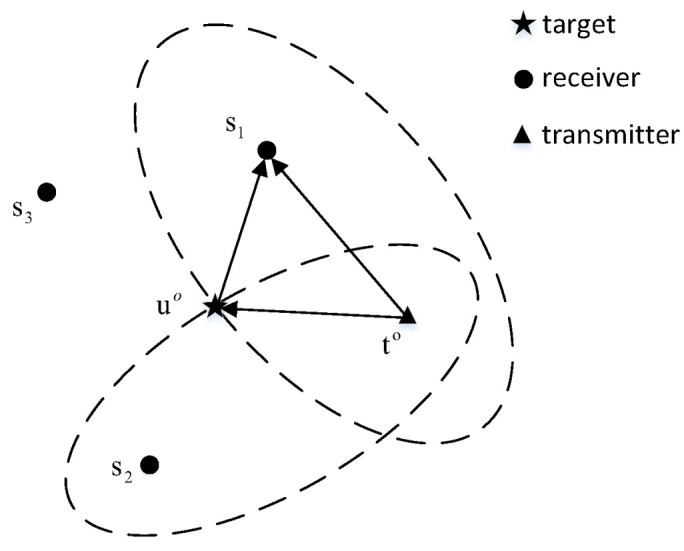
Illustration of elliptic localization.

Let us consider an elliptic localization system for locating an unknown target, in which one transmitter and *M* receivers are deployed in *p*-dimensional space. As can be seen from Figure 1, the unknown transmitter represented by $t^{o}\in R^{p\times 1}$ sends out the radar signal. Several known receivers $s_{j}\in R^{p\times 1}$ for j=1,⋯,M not only collect the indirect path signal reflected from the interest target denoted by $u^{o}\in R^{p\times 1}$, but receive the direct path signal from the transmitter.

(1) $$r_{i}=∥u^{o}-s_{i}∥+n_{i},\;i=1,\cdots ,M$$

and

(2) $$r_{i}=∥u^{o}-t∥+∥u^{o}-s_{i}∥+n_{r,i},\;i=1,\cdots ,M$$

(3) $$d_{i}=∥t^{o}-s_{i}∥+n_{d,i},\;i=1,\cdots ,M$$

Multiplying by the signal propagation speed, the range measurements of the indirect and direct paths can be expressed by

(4) $$r_{j}=∥u^{o}-t^{o}∥+∥u^{o}-s_{j}∥+n_{r,j},\;j=1,\cdots ,M,$$

and

(5) $$d_{j}=∥t^{o}-s_{j}∥+n_{d,j},\;j=1,\cdots ,M,$$

respectively, where $n_{r,j}$ and $n_{d,j}$ are assumed to be independent zero-mean Gaussian variables with known variance $\sigma_{j}^{2}$ and $\beta_{j}^{2}$, respectively.

## 3. Majorization–Minimization for Elliptic Localization

In this section, we present the proposed iterative algorithm based on the MM principle to tackle the elliptic location problem with an unknown transmitter position. Before we depict the proposed algorithm, we briefly introduce the MM principle.

### 3.1. Majorization–Minimization (MM)

**Figure 2:**
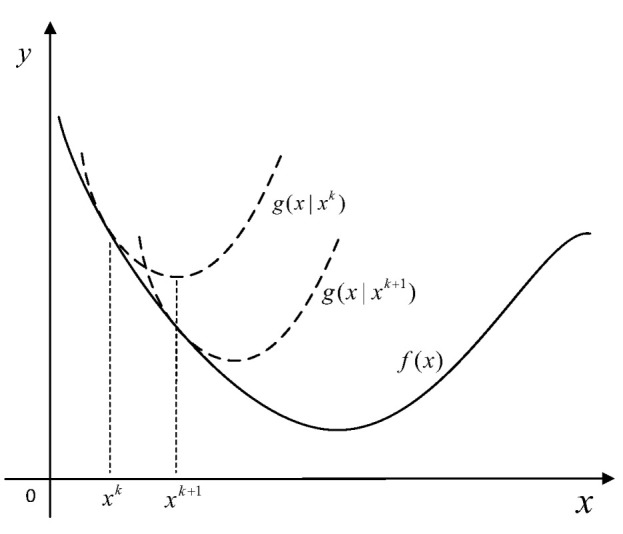
Illustration of MM principle.

The MM procedure is an efficient iterative method for addressing the non-convex problem. As shown in Figure 2, the MM procedure requires constructing a surrogate function ($g(x|x^{k})$) that tightly upper bounds the original objective function (f(x)) at a given point ($x^{k}$), and then minimizes the surrogate function to obtain the next iteration.

As the tight upper bound of the f(x), the $g(x|x^{k})$ need satisfy

(6) $$\begin{array}{c} g(x|x^{k})\geq f(x),\;\forall x,\\ g(x^{k}|x^{k})=f(x^{k}). \end{array}$$

Then in the minimization step, we update x as

(7) $$\begin{array}{c} x^{k+1}=\arg\min_{\begin{array}{c} x \end{array}}g\left(x|x^{k}\right). \end{array}$$

According to (3) and (4), we can simply obtain

(8) $$\begin{array}{c} f\left(x^{k+1}\right)\leq g\left(x^{k+1}|x^{k}\right)\leq g\left(x^{k}|x^{k}\right)=f\left(x^{k}\right). \end{array}$$

It can be readily proved from the inequality in (8) that the original objective function decreases monotonously during the updating process. The construction of the surrogate function is the key problem of the MM algorithm, which will determine the accuracy and complexity of the optimization approach. Interested readers can refer to [34] to acquire more details.

### 3.2. Elliptic Localization Using Single Transmitter

In this subsection, we consider the scenario with one transmitter and apply the MM framework to tackle the problem. From the measurement equations in (4) and (5), an MLE problem for evaluating the target position can be formulated as

(9) $$\begin{array}{c} \min_{\begin{array}{c} u^{o},t^{o} \end{array}}\;\sum_{j=1}^{M}\left(\frac{1}{\sigma_{j}^{2}}{\left(r_{j}-∥u^{o}-t^{o}∥-∥u^{o}-s_{j}∥\right)}^{2}+\frac{1}{\beta_{j}^{2}}{\left(d_{j}-∥t^{o}-s_{j}∥\right)}^{2}\right). \end{array}$$

Combining $u^{o}$ and $t^{o}$ with θ, the cost function can be further rewritten as

(10) $$\begin{array}{c} \min_{\begin{array}{c} \theta \end{array}}\sum_{j=1}^{M}\left(\frac{1}{\sigma_{j}^{2}}{\left(r_{j}-∥D_{1}\theta ∥-∥D_{2}\theta -s_{j}∥\right)}^{2}+\frac{1}{\beta_{j}^{2}}{\left(d_{j}-∥D_{3}\theta -s_{j}∥\right)}^{2}\right), \end{array}$$

where

(11) $$\begin{array}{ccccc} \theta & ={\left[\begin{array}{cc} u^{oT}, & t^{oT} \end{array}\right]}^{T}, & \;\;D_{1} & =\left[I_{p\times p},\;-I_{p\times p}\right], & \\ \;\;D_{2} & =\left[\begin{array}{cc} I_{p\times p}, & O_{p\times p} \end{array}\right], & D_{3} & =\left[O_{p\times p},\;I_{p\times p}\right]. \end{array}$$

Then, expanding the square of (10) and ignoring the constant terms, i.e., $\sum_{j=1}^{M}\frac{1}{\sigma_{j}^{2}}r_{j}^{2}$ and $\sum_{j=1}^{M}\frac{1}{\beta_{j}^{2}}d_{j}^{2}$, we can obtain

(12) $$\begin{array}{c} \min_{\begin{array}{c} \theta \end{array}}\;\sum_{j=1}^{M}(\frac{1}{\sigma_{j}^{2}}\left({\left(∥D_{1}\theta ∥+∥D_{2}\theta -s_{j}∥\right)}^{2}-2r_{j}∥D_{1}\theta ∥-2r_{j}∥D_{2}\theta -s_{j}∥\right)\\ \;\;+\frac{1}{\beta_{j}^{2}}\left({∥D_{3}\theta -s_{j}∥}^{2}-2d_{j}∥D_{3}\theta -s_{j}∥\right)). \end{array}$$

As can be observed, the problem is non-convex. Therefore, we can exploit the MM principle to develop an iterative method to solve it. As mentioned before, constructing a tight surrogate function is the key problem for the MM method [35]. Next, we shall derive the surrogate function by inequality scaling.

According to the inequality of arithmetic and geometric means, we known

(13) $$\begin{array}{c} \sqrt{ab}\leq \frac{a+b}{2},\;a\geq 0,\;b\geq 0. \end{array}$$

Plugging $a={\left(\frac{x}{\tilde{x}}\right)}^{2}$ and $b={\left(\frac{y}{\tilde{y}}\right)}^{2}$ ($x,\tilde{x},y,\tilde{y}$ are all positive numbers) into the above inequality, we can get

(14) $$\begin{array}{c} 2xy\leq \tilde{x}\tilde{y}\left({\left(\frac{x}{\tilde{x}}\right)}^{2}+{\left(\frac{y}{\tilde{y}}\right)}^{2}\right)=\frac{\tilde{y}x^{2}}{\tilde{x}}+\frac{\tilde{x}y^{2}}{\tilde{y}}. \end{array}$$

Then we can easily obtain

(15) $$\begin{array}{c} {\left(x+y\right)}^{2}\leq \left(1+\frac{\tilde{y}}{\tilde{x}}\right)x^{2}+\left(1+\frac{\tilde{x}}{\tilde{y}}\right)y^{2}. \end{array}$$

Using the above inequality, for any given $\theta^{k}$, the function ${\left(∥D_{1}\theta ∥+∥D_{2}\theta -s_{j}∥\right)}^{2}$ can be upper bounded as

(16) $$\begin{array}{c} \sum_{j=1}^{M}\frac{1}{\sigma_{j}^{2}}{\left(∥D_{1}\theta ∥+∥D_{2}\theta -s_{j}∥\right)}^{2}\leq \sum_{j=1}^{M}\frac{1}{\sigma_{j}^{2}}\left(v_{j}{∥D_{1}\theta ∥}^{2}+w_{j}{∥D_{2}\theta -s_{j}∥}^{2}\right), \end{array}$$

where

(17) $$\begin{array}{c} v_{j}=1+\frac{∥D_{2}\theta^{k}-s_{j}∥}{∥D_{1}\theta^{k}∥},w_{j}=1+\frac{∥D_{1}\theta^{k}∥}{∥D_{2}\theta^{k}-s_{j}∥}. \end{array}$$

According to the Cauchy–Schwartz inequality, for any given $\theta^{k}$, these functions $∥D_{1}\theta ∥$, $∥D_{2}\theta -s_{j}∥$ and $∥D_{3}\theta -s_{j}∥$ can be upper bounded as

(18) $$\begin{array}{c} -\sum_{j=1}^{M}\frac{1}{\sigma_{j}^{2}}r_{j}∥D_{1}\theta ∥\leq -\sum_{j=1}^{M}\frac{1}{\sigma_{j}^{2}}r_{j}x^{T}\theta , \end{array}$$

(19) $$\begin{array}{c} -\sum_{j=1}^{M}\frac{1}{\sigma_{j}^{2}}r_{j}∥D_{2}\theta -s_{j}∥\leq -\sum_{j=1}^{M}\frac{1}{\sigma_{j}^{2}}r_{j}y_{j}^{T}\left(D_{2}\theta -s_{j}\right), \end{array}$$

and

(20) $$\begin{array}{c} -\sum_{j=1}^{M}\frac{1}{\beta_{j}^{2}}d_{j}∥D_{3}\theta -s_{j}∥\leq -\sum_{j=1}^{M}\frac{1}{\beta_{j}^{2}}d_{j}z_{j}^{T}\left(D_{3}\theta -s_{j}\right), \end{array}$$

respectively, where

(21) $$\begin{array}{c} x=\frac{D_{1}^{T}D_{1}\theta^{k}}{∥D_{1}\theta^{k}∥},y_{j}=\frac{D_{2}\theta^{k}-s_{j}}{∥D_{2}\theta^{k}-s_{j}∥},z_{j}=\frac{D_{3}\theta^{k}-s_{j}}{∥D_{3}\theta^{k}-s_{j}∥}. \end{array}$$

Note that the denominators of these functions are not zero, and this situation can be avoided in practical scenario.

Using these inequalities in (16) and (18)–(20), we get the following surrogate function for the cost function in (12) at ${\left(k+1\right)}^{th}$ iteration:

(22) $$\begin{array}{cc} g\left(\theta |\theta^{k}\right)= & \sum_{j=1}^{M}(\frac{1}{\sigma_{j}^{2}}\left(v_{j}{∥D_{1}\theta ∥}^{2}+w_{j}{∥D_{2}\theta -s_{j}∥}^{2}-2r_{j}x^{T}\theta -2r_{j}y_{j}^{T}\left(D_{2}\theta -s_{j}\right)\right)\\ & +\frac{1}{\beta_{j}^{2}}\left({∥D_{3}\theta -s_{j}∥}^{2}-2d_{j}z_{j}^{T}\left(D_{3}\theta -s_{j}\right)\right)). \end{array}$$

We can find that the surrogate function satisfies the conditions in (6), i.e., $g\left(\theta |\theta^{k}\right)\geq f\left(\theta \right),\;\forall \theta ,g\left(\theta^{k}|\theta^{k}\right)=f\left(\theta^{k}\right)$. Therefore, it is one of the tight upper bounds of the original cost function (f(θ)) at the point ($\theta^{k}$). Then, minimizing the surrogate function, the updating process is expressed as

(23) $$\begin{array}{cc} \theta^{k+1}=\min_{\begin{array}{c} \theta \end{array}}\sum_{j=1}^{M} & g\left(\theta |\theta^{k}\right). \end{array}$$

It can be simply seen that the surrogate function is convex. By taking the derivative of (22) and letting it equal zero, we can get

(24) $$\begin{array}{c} \theta^{k+1}=Q^{-1}\sum_{j=1}^{M}\left(\frac{1}{\sigma_{j}^{2}}\left(r_{j}\left(x^{T}+D_{2}^{T}y_{j}\right)+w_{j}D_{2}^{T}s_{j}\right)+\frac{1}{\beta_{j}^{2}}\left(D_{3}^{T}s_{j}-d_{j}D_{3}^{T}z_{j}\right)\right), \end{array}$$

where

(25) $$\begin{array}{c} Q=\sum_{j=1}^{M}\left(\frac{1}{\sigma_{j}^{2}}\left(v_{j}D_{1}^{T}D_{1}+w_{j}D_{2}^{T}D_{2}\right)+\frac{1}{\beta_{j}^{2}}D_{3}^{T}D_{3}\right). \end{array}$$

A limit on the number of iterations, $K_{max}$, should be applied for practical scenarios. The criterion to stop the iteration is when

(26) $$\begin{array}{c} ∥\theta^{k+1}-\theta^{k}∥\leq \epsilon ,k=0,1,\cdots ,K_{max}, \end{array}$$

or when the time of iteration attains $K_{max}$, where ϵ is a preset threshold. Then, the estimated results of the target and transmitter positions are expressed as

(27) $$\begin{array}{c} u^{*}=\theta_{\left(1:p\right)}^{*},\;\;t^{*}=\theta_{\left(p+1:2p\right)}^{*}, \end{array}$$

respectively, where $\theta^{*}$ is the final result of iterations. In Algorithm 1, we give the relevant pseudo code.
**Algorithm 1:** Elliptic Localization using single transmitter
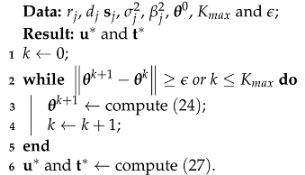


### 3.3. Elliptic Localization Using Multiple Transmitters with Receiver Position Errors

It is common in practical applications, especially in sonar/radar, to deploy multiple transmitters for improving performance. Moreover, due to imperfections in GPS precision, the available receiver positions may present errors. Ignoring these errors can generate significant performance degradation [20]. In the subsection, we further apply the MM method to tackle the general scenario where both multiple unknown transmitters and receiver position errors are considered.

The unknown position of the *i*-th transmitter is represented by $t_{i}^{o}\in R^{p\times 1},\;i=1,\cdots ,N$. Then, the range measurements of the indirect and direct paths are expressed by

(28) $$\begin{array}{c} r_{ij}=∥u^{o}-t_{i}^{o}∥+∥u^{o}-s_{j}^{o}∥+n_{r,ij},\;i=1,\cdots ,N,\;j=1,\cdots ,M, \end{array}$$

and

(29) $$d_{ij}=∥t_{i}^{o}-s_{j}^{o}∥+n_{d,ij},\;i=1,\cdots ,N,\;j=1,\cdots ,M,$$

respectively. Furthermore, let ${\tilde{s}}_{j}\in R^{p\times 1},\;j=1,\cdots ,M$ be the available position of the *j*-th receiver. The true position $s_{j}^{o}$ is not known and always modeled as

(30) $$\begin{array}{c} {\tilde{s}}_{j}=s_{j}^{o}+\Delta s_{j},\;j=1,2,\cdots ,M, \end{array}$$

where $\Delta s_{j}$ is the position error of $s_{j}$ that obeys the zero-mean Gaussian distribution with known covariance $\gamma_{j}^{2}I_{p\times p}$.

From the measurement equations in (28)–(30), an MLE problem for evaluating the target position can be formulated as

(31) $$\begin{array}{c} \min_{\begin{array}{c} u^{o},t_{1}^{o},\cdots ,t_{N}^{o},s_{1}^{o},\cdots ,s_{M}^{o} \end{array}}\;\sum_{i=1}^{N}\sum_{j=1}^{M}\left(\frac{1}{\sigma_{ij}^{2}}{\left(r_{ij}-∥u^{o}-t_{i}^{o}∥-∥u^{o}-s_{j}^{o}∥\right)}^{2}+\frac{1}{\beta_{ij}^{2}}{\left(d_{ij}-∥t_{i}^{o}-s_{j}^{o}∥\right)}^{2}\right)+\sum_{j=1}^{M}\frac{1}{\gamma_{j}^{2}}{∥{\tilde{s}}_{j}-s_{j}^{o}∥}^{2} \end{array}$$

Using a vector θ to combine these unknown variables, we can obtain

(32) $$\begin{array}{c} \min_{\begin{array}{c} \theta \end{array}}\sum_{i=1}^{N}\sum_{j=1}^{M}\left(\frac{1}{\sigma_{ij}^{2}}{\left(r_{ij}-∥H_{i}\theta ∥-∥P_{j}\theta ∥\right)}^{2}+\frac{1}{\beta_{ij}^{2}}{\left(d_{ij}-∥Q_{ij}\theta ∥\right)}^{2}\right)+\sum_{j=1}^{M}\frac{1}{\gamma_{j}^{2}}{∥{\tilde{s}}_{j}-F_{j}\theta ∥}^{2}, \end{array}$$

where,

(33) $$\begin{array}{cc} \; & \theta ={\left[\begin{array}{ccccccc} u^{T} & t_{1}^{oT} & \cdots & t_{N}^{oT} & s_{1}^{oT} & \cdots & s_{M}^{oT} \end{array}\right]}^{T},\\ \; & F_{j}=\left[\begin{array}{ccc} O_{p\times (N+j)p} & I_{p\times p} & O_{p\times (M-j)p} \end{array}\right],\;j=1,\cdots ,M,\\ \; & H_{i}=\left[\begin{array}{cccc} I_{p\times p} & O_{p\times (i-1)p} & -I_{p\times p} & O_{p\times (N+M-i)p} \end{array}\right],\;i=1,\cdots ,N,\\ \; & P_{j}=\left[\begin{array}{cccc} I_{p\times p} & O_{p\times (N+j-1)p} & -I_{p\times p} & O_{p\times (M-j)p} \end{array}\right],\;j=1,\cdots ,M,\\ \; & Q_{ij}=\left[\begin{array}{ccccc} O_{p\times ip} & I_{p\times p} & O_{p\times (N+i-j-1)p} & -I_{p\times p} & O_{p\times (M-j)p} \end{array}\right],\;i=1,\cdots ,N,\;j=1,\cdots ,M. \end{array}$$

Then, expanding the square of (32) and ignoring the constant terms, i.e., $\sum_{i=1}^{N}\sum_{j=1}^{M}\frac{1}{\sigma_{ij}^{2}}r_{ij}^{2}$ and $\sum_{i=1}^{N}\sum_{j=1}^{M}\frac{1}{\beta_{ij}^{2}}d_{ij}^{2}$, we can obtain

(34) $$\begin{array}{c} \min_{\begin{array}{c} \theta \end{array}}\sum_{i=1}^{N}\sum_{j=1}^{M}\left(\frac{1}{\sigma_{ij}^{2}}\left({\left(∥H_{i}\theta ∥+∥P_{j}\theta ∥\right)}^{2}-2r_{ij}∥H_{i}\theta ∥-2r_{ij}∥P_{j}\theta ∥\right)+\frac{1}{\beta_{ij}^{2}}\left({∥Q_{ij}\theta ∥}^{2}-2d_{ij}∥Q_{ij}\theta ∥\right)\right)+\\ \;\;\sum_{j=1}^{M}\frac{1}{\gamma_{j}^{2}}{∥{\tilde{s}}_{j}-F_{j}\theta ∥}^{2}. \end{array}$$

Similarly, we shall utilize the MM principle to account for the non-convex problem as well. As already mentioned, we shall utilize these inequalities mentioned in the previous subsection to construct the surrogate function.

Using the inequality in (15), for any given $\theta^{k}$, the function ${\left(∥H_{i}\theta ∥+∥P_{j}\theta ∥\right)}^{2}$ can be upper bounded as

(35) $$\begin{array}{c} \sum_{i=1}^{N}\sum_{j=1}^{M}\frac{1}{\sigma_{ij}^{2}}{\left(∥H_{i}\theta ∥+∥P_{j}\theta ∥\right)}^{2}\leq \sum_{i=1}^{N}\sum_{j=1}^{M}\frac{1}{\sigma_{ij}^{2}}\left({\tilde{v}}_{ij}{∥H_{i}\theta ∥}^{2}+{\tilde{w}}_{ij}{∥P_{j}\theta ∥}^{2}\right), \end{array}$$

where

(36) $$\begin{array}{c} {\tilde{v}}_{ij}=1+\frac{∥P_{j}\theta^{k}∥}{∥H_{i}\theta^{k}∥},{\tilde{w}}_{ij}=1+\frac{∥H_{i}\theta^{k}∥}{∥P_{j}\theta^{k}∥}. \end{array}$$

According to the Cauchy–Schwartz inequality, for any given $\theta^{k}$, these functions $∥H_{i}\theta ∥$, $∥P_{j}\theta ∥$ and $∥Q_{ij}\theta ∥$ can be upper bounded as

(37) $$\begin{array}{c} -\sum_{i=1}^{N}\sum_{j=1}^{M}\frac{1}{\sigma_{ij}^{2}}r_{ij}∥H_{i}\theta ∥\leq -\sum_{i=1}^{N}\sum_{j=1}^{M}\frac{1}{\sigma_{ij}^{2}}r_{ij}{\tilde{x}}_{i}^{T}\theta , \end{array}$$

(38) $$\begin{array}{c} -\sum_{i=1}^{N}\sum_{j=1}^{M}\frac{1}{\sigma_{ij}^{2}}r_{ij}∥P_{j}\theta ∥\leq -\sum_{i=1}^{N}\sum_{j=1}^{M}\frac{1}{\sigma_{ij}^{2}}r_{ij}{\tilde{y}}_{j}^{T}\theta , \end{array}$$

and

(39) $$\begin{array}{c} -\sum_{i=1}^{N}\sum_{j=1}^{M}\frac{1}{\beta_{ij}^{2}}d_{ij}∥Q_{ij}\theta ∥\leq -\sum_{i=1}^{N}\sum_{j=1}^{M}\frac{1}{\beta_{ij}^{2}}d_{ij}{\tilde{z}}_{ij}^{T}\theta , \end{array}$$

respectively, where

(40) $$\begin{array}{c} {\tilde{x}}_{i}=\frac{H_{i}^{T}H_{i}\theta^{k}}{∥H_{i}\theta^{k}∥},\;{\tilde{y}}_{j}=\frac{P_{j}^{T}P_{j}\theta^{k}}{∥P_{j}\theta^{k}∥},\;{\tilde{z}}_{ij}=\frac{Q_{ij}^{T}Q_{ij}\theta^{k}}{∥Q_{ij}\theta^{k}∥}. \end{array}$$

Note that the denominators of these functions are not zero as well.

Using these inequalities in (35) and (37)–(39), we get the following surrogate function for the cost function in (34) at ${\left(k+1\right)}^{th}$ iteration:

(41) $$\begin{array}{cc} g\left(\theta |\theta^{k}\right)= & \sum_{i=1}^{N}\sum_{j=1}^{M}\left(\frac{1}{\sigma_{ij}^{2}}\left({\tilde{v}}_{ij}{∥H_{i}\theta ∥}^{2}+{\tilde{w}}_{ij}{∥P_{j}\theta ∥}^{2}-2r_{ij}\left({\tilde{x}}_{i}^{T}\theta -{\tilde{y}}_{j}^{T}\theta \right)\right)+\frac{1}{\beta_{ij}^{2}}\left({∥Q_{ij}\theta ∥}^{2}-2d_{ij}{\tilde{z}}_{ij}^{T}\theta \right)\right)+\\ & \sum_{j=1}^{M}\frac{1}{\gamma_{j}^{2}}{∥{\tilde{s}}_{j}-F_{j}\theta ∥}^{2}. \end{array}$$

Minimizing the surrogate function, the updating process can be expressed as

(42) $$\begin{array}{c} \theta^{k+1}=W^{-1}\left(\sum_{i=1}^{N}\sum_{j=1}^{M}\left(\frac{1}{\sigma_{ij}^{2}}r_{ij}\left({\tilde{x}}_{i}+{\tilde{y}}_{j}\right)+\frac{1}{\beta_{ij}^{2}}d_{ij}{\tilde{z}}_{ij}\right)+\sum_{j=1}^{M}\frac{1}{\gamma_{j}^{2}}F_{i}^{T}{\tilde{s}}_{i}\right), \end{array}$$

where

(43) $$\begin{array}{c} W=\sum_{i=1}^{N}\sum_{j=1}^{M}\left(\frac{1}{\sigma_{ij}^{2}}\left({\tilde{v}}_{ij}H_{i}^{T}H_{i}+{\tilde{w}}_{ij}P_{j}^{T}P_{j}\right)+\frac{1}{\beta_{ij}^{2}}Q_{ij}^{T}Q_{ij}\right)+\sum_{j=1}^{M}\frac{1}{\gamma_{j}^{2}}F_{j}^{T}F_{j}. \end{array}$$

The criterion to stop the iteration is same to the previous subsection. Then, the estimated results of the target, transmitter, and receiver positions are expressed as

(44) $$\begin{array}{c} u^{*}=\theta_{\left(1:p\right)}^{*},\;t_{i}^{*}=\theta_{\left(pi+1:p(i+1)\right)}^{*},\;s_{j}^{*}=\theta_{\left(p(N+j)+1:p(N+j+1)\right)}^{*},\;i=1,\cdots ,N,\;j=1,\cdots ,M, \end{array}$$

respectively, where $\theta^{*}$ is the final result of iterations. As we can see from (44), different from the existing approaches in [19,20], the proposed method can not only estimate the positions of target and transmitters, but also calibrate the erroneous receiver positions.

### 3.4. Computational Complexity and Convergence of the Proposed Algorithm

We first analyze the computational complexity of the proposed methods. In each iteration, the complexity is mainly dominated by computing (24) for the single transmitter case and (42) for the multiple transmitters case. Hence, their complexities are on the order of $O(M^{2})$ and $O({\left(N+M\right)}^{4})$, respectively. Moreover, the average computation time will be shown in the next section.

Next, we shall discuss the convergence of the proposed algorithm. Since the algorithm is based on the MM framework, it is seen from (8) that the objective function monotonically decreases with the iteration. Moreover, the surrogate function at each iteration is convex, so we can easily get its solution $\theta^{k+1}$ in ${\left(k+1\right)}^{th}$ iteration that makes

(45) $$\begin{array}{c} {\left.\frac{\partial g(\theta |\theta^{k})}{\partial \theta }\right|}_{\theta =\theta^{k+1}}=0 \end{array}$$

and

(46) $$\begin{array}{c} g\left(\theta^{k+1}|\theta^{k}\right)\leq g\left(\theta |\theta^{k}\right). \end{array}$$

Since ${\left.\frac{\partial f(\theta )}{\partial \theta }\right|}_{\theta =\theta^{k}}={\left.\frac{\partial g(\theta |\theta^{k})}{\partial \theta }\right|}_{\theta =\theta^{k}}$, taking k→∞ gives

(47) $$\begin{array}{c} {\left.\frac{\partial f(\theta )}{\partial \theta }\right|}_{\theta =\theta^{\infty }}={\left.\frac{\partial g(\theta |\theta^{\infty })}{\partial \theta }\right|}_{\theta =\theta^{\infty }}=0 \end{array}$$

Hence, through the proposed algorithm, any given initial value will constantly approach the stationary point of the object function.

## 4. Simulation Results

In this section, numerical simulations are conducted to evaluate the performance of the proposed method in 2-D for ease of illustration. The proposed method is compared with the SDP method [19] (labeled as “SDP”), the two-stage WLS method [20] (labeled as “TSWLS”) and the Gauss–Newton method (labeled as “Gauss–Newton”, the derivation is shown in Appendix A). Moreover, the CRLB is also included as a benchmark. Owing to many unknown variables in the objective function, we choose to initialize θ by successive estimation. The successive estimation can be regarded as a TOA localization problem (based on direct paths) and an elliptic localization problem (based on indirect paths). The TOA localization problem is solved to evaluate the transmitter position by the LS method in [36]. Then, using the evaluated transmitter position, the elliptic localization problem is solved to evaluate the target position by the LS method in [28]. Meanwhile, the receiver position errors are not considered. The noise variances of the indirect and direct paths are set to the same value (represented by $\sigma^{2}$). The maximum number of iterations is set to $K_{max}=4000$ and the predetermined threshold is set as $\epsilon =10^{-3}$.

The root mean square error (RMSE) is utilized to evaluate the accuracy of these algorithms, which is expressed as

(48) $$\begin{array}{c} \;\mathrm{RMSE}\left(\eta^{*}\right)=\sqrt{\frac{1}{GL}\sum_{g=1}^{G}\sum_{l=1}^{L}∥\eta_{gl}^{*}-\eta^{o}∥}, \end{array}$$

where *G* are the number of geometry configurations and *L* is the times of Monte Carlo (MC) runs for each geometry configuration. $\eta_{gl}^{*}$ is the estimated result in the *l*-th MC run for the *g*-th configuration. *G* and *L* are adopted as 10 and 500 in the simulations.

Next, we shall test those algorithms in the single transmitter and multiple transmitters cases, respectively.

### 4.1. Single Transmitter Case

**Figure 3:**
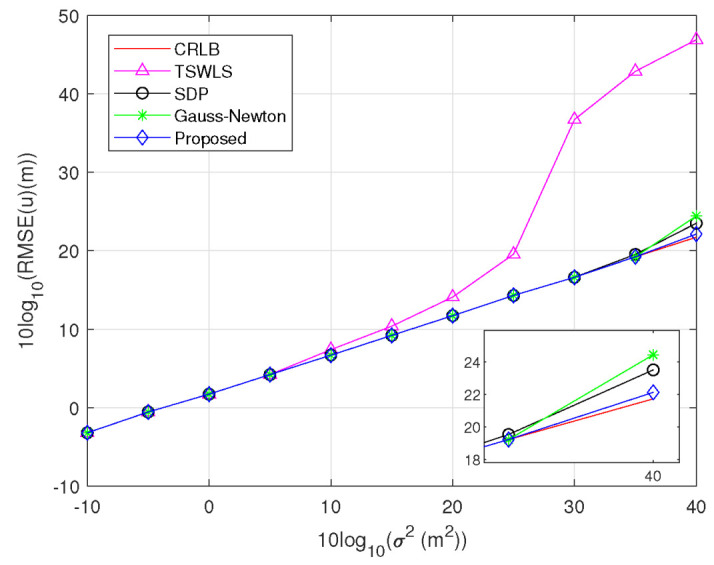
RMSE comparison for estimating target position.

**Figure 4:**
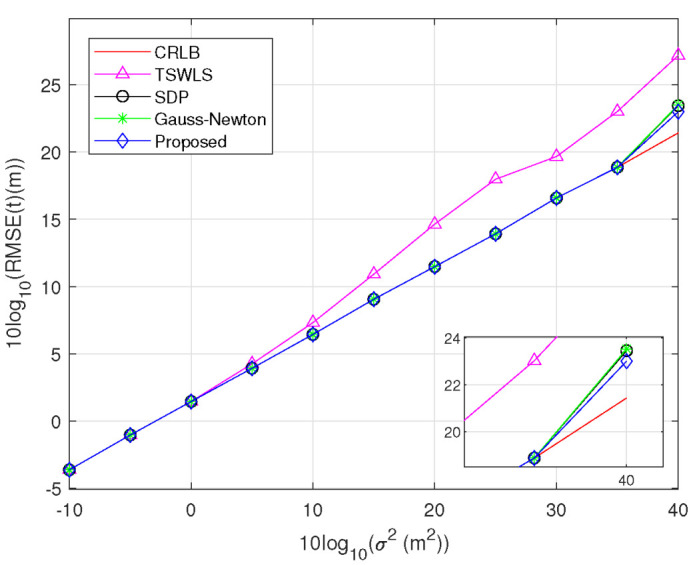
RMSE comparison for estimating target position.

*Scenario 1:* One transmitter and four receivers are uniformly deployed in the region $\left(-4,4\right)\times \left(-4,4\right)\;{\mathrm{km}}^{2}$ to locate a target at $u^{o}={\left[2,1\right]}^{T}$ km. The estimated results of the target position are displayed in Figure 3. We find that the RMSE of the TSWLS method attains the CRLB accuracy when $10\log_{10}\left(\sigma^{2}\right)\leq 5\;\mathrm{dB}m^{2}$, whereas deviates from the CRLB when $10\log_{10}\left(\sigma^{2}\right)\geq 10\;\mathrm{dB}m^{2}$. This may be due to the *threshold effect* of the TSWLS method. By contrast, the proposed method, SDP method and Gauss–Newton method show better threshold behavior, and the first has a higher performance because its algorithm is based on a more accurate problem formulation. Figure 4 displays the estimated results of the transmitter position. It is observed that the RMSEs of the proposed method, SDP method and Gauss–Newton method attain the optimal performance when $10\log_{10}\left(\sigma^{2}\right)\leq 35\;\mathrm{dB}m^{2}$, and slightly deviates from the CRLB at $10\log_{10}\left(\sigma^{2}\right)=40\;\mathrm{dB}m^{2}$.

**Figure 5:**
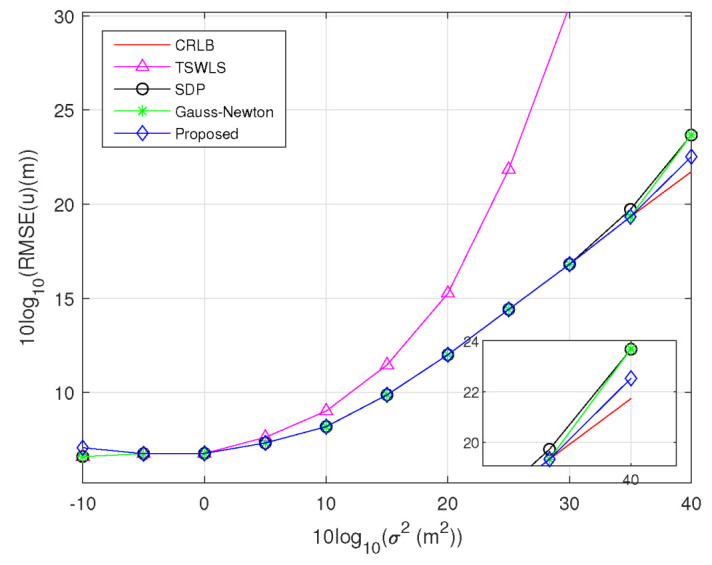
RMSE comparison for estimating target position at $\sigma_{s}^{2}=1\;m^{2}$.

*Scenario 2:* In this scenario, receiver position errors are considered to be present. Assuming the covariance matrix of the receiver position errors $Q_{s}=\sigma_{s}^{2}J$, where $\sigma_{s}^{2}$ relates to the sensor position error power and $J=\mathrm{diag}\left[5,20,15,10\right]⊗I_{2\times 2}$. The deployment of the target, transmitter, and receiver is the same as that in Scenario 1. Keeping $\sigma_{s}^{2}$ at 1 $m^{2}$, Figure 5 confirms that the RMSE of the TSWLS method attains the CRLB accuracy when $10\log_{10}\left(\sigma^{2}\right)\leq 5\;\mathrm{dB}m^{2}$ and deviates from the CRLB when $10\log_{10}\left(\sigma^{2}\right)\geq 10\;\mathrm{dB}m^{2}$ much earlier than the other methods. When $10\log_{10}\left(\sigma^{2}\right)=40\;\mathrm{dB}m^{2}$, the proposed methods still performs better than the SDP method and Gauss–Newton method.

**Figure 6:**
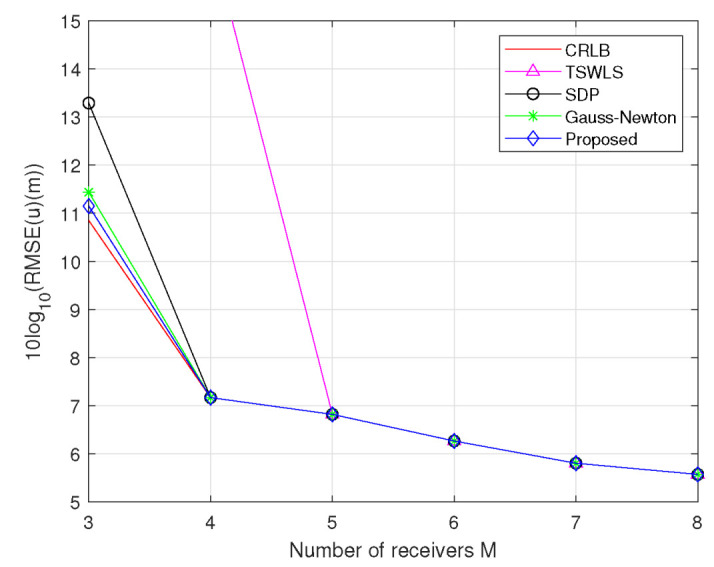
RMSE comparison for estimating target position at $\sigma^{2}=10\;m^{2}$ and $\sigma_{s}^{2}=1\;m^{2}$.

*Scenario 3:* In this scenario, the number of receiver *M* increases from 3 to 8. We let $\sigma^{2}=10$ $m^{2}$ and $Q_{s}=\sigma_{s}^{2}J_{M}$, where $\sigma_{s}^{2}=1\;m^{2}$, $J_{M}=J_{\left(1:2M,1:2M\right)}$ for M=4,5,⋯,8 and $J=\mathrm{diag}\left[5,20,35,10,25,30,15,40\right]⊗I_{2\times 2}$. The deployment of the actual coordinates of the target, transmitter, and receiver is the same as that in Scenario 1. As can be seen in Figure 6, the performance of these methods improves as the number of receivers increases. The RMSE of the TSWLS method deviates from the CRLB accuracy when M≤4. The proposed method, SDP method and Gauss–Newton method attain the CRLB accuracy at the entire tested range except for M=3. For M=3, the SDP method obviously deviates from CRLB; however, the proposed method and Gauss–Newton method just slightly deviate from CRLB, and the former has better performance.

### 4.2. Multiple Transmitters Case

**Figure 7:**
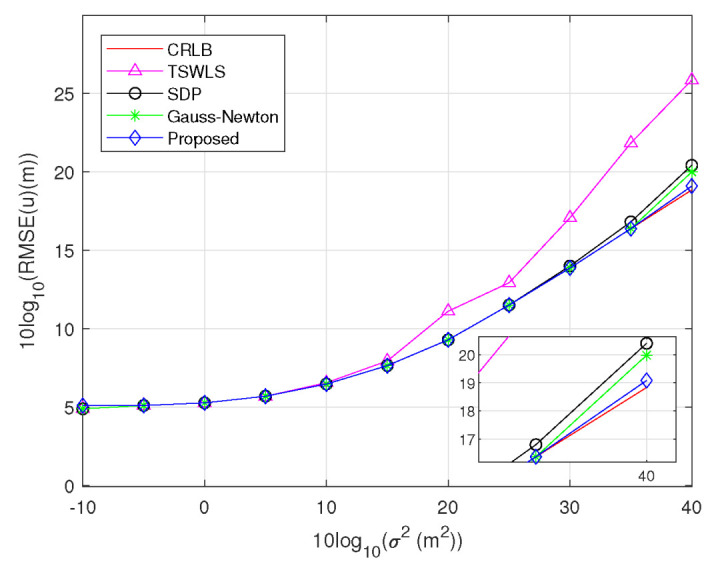
RMSE comparison for estimating target position at $\sigma_{s}^{2}=1\;m^{2}$ using three transmitters.

*Scenario 4:* Multiple transmitters and receiver position errors are simultaneously considered in this Scenario. Three transmitters and four receivers are used, and the other parameter settings are the same as that in Scenario 2. As can be seen in Figure 7, the observations are similar to those in Figure 5, whereas the RMSEs are lower at the same $\sigma^{2}$. This is caused by using multiple transmitters.

**Figure 8:**
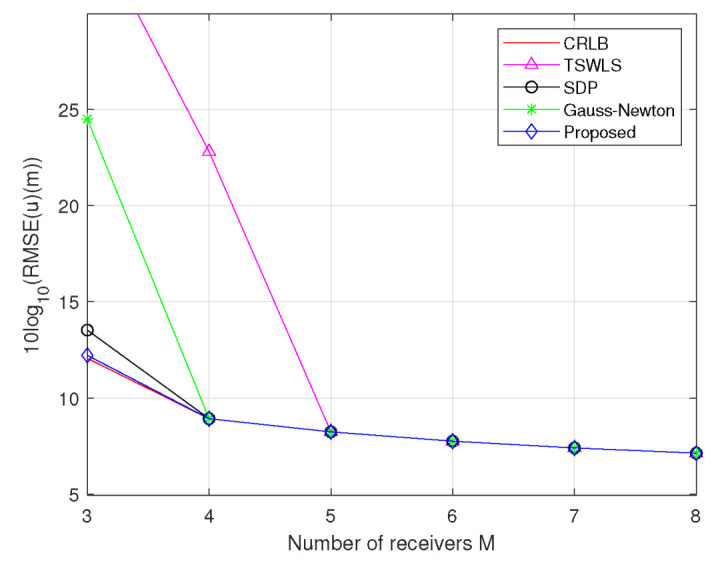
RMSE comparison for estimating target position at $\sigma^{2}=100\;m^{2}$ and $\sigma_{s}^{2}=1\;m^{2}$ using three transmitters.

*Scenario 5:* Fixing three transmitters, the number of receiver *M* increases from 3 to 8. We let $\sigma^{2}=100$ $m^{2}$ and $Q_{s}=\sigma_{s}^{2}J_{M}$ with $\sigma_{s}^{2}=1\;m^{2}$, and the other parameter settings are the same as that in Scenario 3. As we can see in Figure 8, the performance of these methods improves with the rise of the receiver number. Moreover, the TSWLS method deviates from the CRLB accuracy when M≤4 and the SDP method and Gauss–Newton method deviate from the CRLB accuracy at M=3. By contrast, the proposed method can approach the CRLB accuracy at the whole tested noise level.

Finally, the average CPU time of the methods in Scenario 1 and Scenario 4 is shown in Table 1. The simulation data is obtained by utilizing a PC with Intel Core i7 3.2 GHz processor. As reported in Table 1, the computational complexity of these methods increases as the measurement model becomes more complex. Furthermore, the computational complexity of the proposed method is higher than that of the TSWLS method and Gauss–Newton method, and lower than that of the SDP method.

## 5. Conclusions

In this paper, we investigate an elliptic localization problem when the transmitter position is unavailable. An MM method is developed to jointly estimate the target and transmitter positions. Subsequently, the MM method is further extended to account for scenarios where both multiple unknown transmitters and receiver position errors are considered. Simulation results demonstrate that the proposed method outperforms state-of-the-art methods and the erroneous receiver positions can also be calibrated.

## Figures and Tables

**Table 1:** Avg. CPU time [ms] for methods.

	Scenarios	1	4
Algorithms			
TSWLS		0.38	0.84
SDP		551.6	695.4
Gauss–Newton		0.23	1.6
Proposed		10.1	157.7

## Data Availability

Not applicable.

## Sample Availability

The codes of simulations are available from the authors via email ybzou@stu.edu.cn.

## Abbreviations

The following abbreviations are used in this manuscript:

WSN	Wireless sensor network
TOA	Time-of-arrival
TDOA	Time-difference-of-arrival
RSS	Received signal strength
AOA	Angle-of-arrival
MIMO	Multiple-input-multiple-output
TSWLS	Two-step weighted least squares
SDP	Semidefinite programming
SDR	Semidefinite relaxation
MLE	Maximum likelihood
MM	Majorization–minimization
GTRS	Generalized trust region sub-problems
CRLB	Cramer–Rao lower bound
RMSE	Root mean-square error

## Appendix A. Gauss–Newton Method for Elliptic Localization in Absence of Transmitter Position

### Appendix A.1. Elliptic Localization Using Single Transmitter

In this appendix, the Gauss–Newton method for elliptic localization using single transmitter is derived, and the derivation process is similar to [37]. Rewriting the MLE problem in (10)

(A1) $$\begin{array}{c} \min_{\begin{array}{c} \theta \end{array}}\sum_{j=1}^{M}\left(\frac{1}{\sigma_{j}^{2}}{\left(r_{j}-∥D_{1}\theta ∥-∥D_{2}\theta -s_{j}∥\right)}^{2}+\frac{1}{\beta_{j}^{2}}{\left(d_{j}-∥D_{3}\theta -s_{j}∥\right)}^{2}\right) \end{array}$$

The first-order Taylor series expansions of $∥D_{1}\theta ∥$, $∥D_{2}\theta -s_{j}∥$ and $∥D_{3}\theta -s_{j}∥$ are

(A2a) $$\begin{array}{cc} \; & ∥D_{1}\theta ∥\approx ∥D_{1}\theta^{0}∥+\frac{{\left(D_{1}^{T}D_{1}\theta^{0}\right)}^{T}\left(\theta -\theta^{0}\right)}{∥D_{1}\theta^{0}∥}, \end{array}$$

(A2b) $$\begin{array}{cc} \; & ∥D_{2}\theta -s_{j}∥\approx ∥D_{2}\theta^{0}-s_{j}∥+\frac{{\left(D_{2}^{T}\left(D_{2}\theta^{0}-s_{j}\right)\right)}^{T}\left(\theta -\theta^{0}\right)}{∥D_{2}\theta^{0}-s_{j}∥}, \end{array}$$

(A2c) $$\begin{array}{cc} \; & ∥D_{3}\theta -s_{j}∥\approx ∥D_{3}\theta^{0}-s_{j}∥+\frac{{\left(D_{3}^{T}\left(D_{3}\theta^{0}-s_{j}\right)\right)}^{T}\left(\theta -\theta^{0}\right)}{∥D_{3}\theta^{0}-s_{j}∥}, \end{array}$$

respectively. Then, taking (A2) into (A1) results

(A3) $$\begin{array}{cc} \min_{\begin{array}{c} \theta \end{array}} & \sum_{j=1}^{M}(\frac{1}{\sigma_{j}^{2}}{\left(r_{j}-∥D_{1}\theta^{0}∥-∥D_{2}\theta^{0}-s_{j}∥-\left(\frac{{\left(D_{1}^{T}D_{1}\theta^{0}\right)}^{T}}{∥D_{1}\theta^{0}∥}+\frac{{\left(D_{2}^{T}\left(D_{2}\theta^{0}-s_{j}\right)\right)}^{T}}{∥D_{2}\theta^{0}-s_{j}∥}\right)\left(\theta -\theta^{0}\right)\right)}^{2}+\\ & \frac{1}{\beta_{j}^{2}}{\left(d_{j}-∥D_{3}\theta^{0}-s_{j}∥-\frac{{\left(D_{3}^{T}\left(D_{3}\theta^{0}-s_{j}\right)\right)}^{T}\left(\theta -\theta^{0}\right)}{∥D_{3}\theta^{0}-s_{j}∥}\right)}^{2}). \end{array}$$

Let the gradient of cost function in (A3) with θ to zero

(A4) $$\begin{array}{c} \sum_{j=1}^{M}-2(\frac{1}{\sigma_{j}^{2}}\left(r_{j}-∥D_{1}\theta^{0}∥-∥D_{2}\theta^{0}-s_{j}∥-\left(\frac{{\left(D_{1}^{T}D_{1}\theta^{0}\right)}^{T}}{∥D_{1}\theta^{0}∥}+\frac{{\left(D_{2}^{T}\left(D_{2}\theta^{0}-s_{j}\right)\right)}^{T}}{∥D_{2}\theta^{0}-s_{j}∥}\right)\left(\theta -\theta^{0}\right)\right)(\frac{D_{1}^{T}D_{1}\theta^{0}}{∥D_{1}\theta^{0}∥}\\ \;\;+\frac{D_{2}^{T}\left(D_{2}\theta^{0}-s_{j}\right)}{∥D_{2}\theta^{0}-s_{j}∥})+\frac{1}{\beta_{j}^{2}}\left(d_{j}-∥D_{3}\theta^{0}-s_{j}∥-\frac{{\left(D_{3}^{T}\left(D_{3}\theta^{0}-s_{j}\right)\right)}^{T}\left(\theta -\theta^{0}\right)}{∥D_{3}\theta^{0}-s_{j}∥}\right)\frac{D_{3}^{T}\left(D_{3}\theta^{0}-s_{j}\right)}{∥D_{3}\theta^{0}-s_{j}∥})=0 \end{array}$$

Set

(A5a) $$\begin{array}{cc} \; & Q_{r}=\mathrm{diag}\left[\frac{1}{\sigma_{1}^{2}},\ldots ,\frac{1}{\sigma_{M}^{2}}\right],\;Q_{d}=\mathrm{diag}\left[\frac{1}{\beta_{1}^{2}},\ldots ,\frac{1}{\beta_{M}^{2}}\right], \end{array}$$

(A5b) $$\begin{array}{cc} \; & h_{1}={\left[r_{1}-∥D_{1}\theta^{0}∥-∥D_{2}\theta^{0}-s_{1}∥,\ldots ,r_{M}-∥D_{1}\theta^{0}∥-∥D_{2}\theta^{0}-s_{M}∥\right]}^{T}, \end{array}$$

(A5c) $$\begin{array}{cc} \; & h_{2}={\left[d_{1}-∥D_{3}\theta^{0}-s_{1}∥,\ldots ,d_{M}-∥D_{3}\theta^{0}-s_{M}∥\right]}^{T}, \end{array}$$

(A5d) $$\begin{array}{cc} \; & H_{1}=\left[\frac{D_{1}^{T}D_{1}\theta^{0}}{∥D_{1}\theta^{0}∥}+\frac{D_{2}^{T}\left(D_{2}\theta^{0}-s_{1}\right)}{∥D_{2}\theta^{0}-s_{1}∥},\ldots ,\frac{D_{1}^{T}D_{1}\theta^{0}}{∥D_{1}\theta^{0}∥}+\frac{D_{2}^{T}\left(D_{2}\theta^{0}-s_{M}\right)}{∥D_{2}\theta^{0}-s_{M}∥}\right], \end{array}$$

(A5e) $$\begin{array}{cc} \; & H_{2}=\left[\frac{D_{3}^{T}\left(D_{3}\theta^{0}-s_{1}\right)}{∥D_{3}\theta^{0}-s_{1}∥},\ldots ,\frac{D_{3}^{T}\left(D_{3}\theta^{0}-s_{M}\right)}{∥D_{3}\theta^{0}-s_{M}∥}\right] \end{array}$$

Then (A4) can be recast as

(A6) $$\begin{array}{c} H_{1}Q_{r}h_{1}+H_{2}Q_{d}h_{2}=\left(H_{1}Q_{r}H_{1}^{T}+H_{2}Q_{d}H_{2}^{T}\right)\left(\theta -\theta^{0}\right) \end{array}$$

Finally, the updating of θ is

(A7) $$\begin{array}{c} \theta =\theta^{0}+{\left(H_{1}Q_{r}H_{1}^{T}+H_{2}Q_{d}H_{2}^{T}\right)}^{-1}\left(H_{1}Q_{r}h_{1}+H_{2}Q_{d}h_{2}\right) \end{array}$$

### Appendix A.2. Elliptic Localization Using Multiple Transmitters with Receiver Position Errors

In this appendix, the Gauss–Newton method for elliptic localization using multiple transmitters with receiver position errors is derived. Rewriting the MLE problem in (32)

(A8) $$\begin{array}{c} \min_{\begin{array}{c} \theta \end{array}}\sum_{i=1}^{N}\sum_{j=1}^{M}\left(\frac{1}{\sigma_{ij}^{2}}{\left(r_{ij}-∥H_{i}\theta ∥-∥P_{j}\theta ∥\right)}^{2}+\frac{1}{\beta_{ij}^{2}}{\left(d_{ij}-∥Q_{ij}\theta ∥\right)}^{2}\right)+\sum_{j=1}^{M}\frac{1}{\gamma_{j}^{2}}{∥{\tilde{s}}_{j}-F_{j}\theta ∥}^{2}. \end{array}$$

Similarly, thought the first-order Taylor series expansion, we can get

(A9) $$\begin{array}{cc} \min_{\begin{array}{c} \theta \end{array}} & \sum_{i=1}^{N}\sum_{j=1}^{M}(\frac{1}{\sigma_{ij}^{2}}{\left(r_{ij}-∥H_{i}\theta^{0}∥-∥P_{j}\theta^{0}∥-\left(\frac{{\left(H_{i}^{T}H_{i}\theta^{0}\right)}^{T}}{∥H_{i}\theta^{0}∥}+\frac{{\left(P_{j}^{T}P_{j}\theta \right)}^{T}}{∥P_{j}\theta^{0}∥}\right)\left(\theta -\theta^{0}\right)\right)}^{2}+\\ & \frac{1}{\beta_{ij}^{2}}{\left(d_{ij}-∥Q_{ij}\theta^{0}∥-\frac{{\left(Q_{ij}^{T}Q_{ij}\theta^{0}\right)}^{T}\left(\theta -\theta^{0}\right)}{∥Q_{ij}\theta^{0}∥}\right)}^{2})+\sum_{j=1}^{M}\frac{1}{\gamma_{j}^{2}}{∥{\tilde{s}}_{j}-F_{j}\theta ∥}^{2}. \end{array}$$

Let the gradient of objective function in (A9) with θ to zero

(A10) $$\begin{array}{c} \sum_{i=1}^{N}\sum_{j=1}^{M}-2(\frac{1}{\sigma_{ij}^{2}}\left(r_{ij}-∥H_{i}\theta^{0}∥-∥P_{j}\theta^{0}∥-\left(\frac{{\left(H_{i}^{T}H_{i}\theta^{0}\right)}^{T}}{∥H_{i}\theta^{k}∥}+\frac{{\left(P_{j}^{T}P_{j}\theta^{0}\right)}^{T}}{∥P_{j}\theta^{0}∥}\right)\left(\theta -\theta^{0}\right)\right)\left(\frac{H_{i}^{T}H_{i}\theta^{0}}{∥H_{i}\theta^{0}∥}+\frac{P_{j}^{T}P_{j}\theta^{0}}{∥P_{j}\theta^{0}∥}\right)\\ \;\;\;\;+\frac{1}{\beta_{ij}^{2}}\left(d_{ij}-∥Q_{ij}\theta^{0}∥-\frac{{\left(Q_{ij}^{T}Q_{ij}\theta^{0}\right)}^{T}\left(\theta -\theta^{0}\right)}{∥Q_{ij}\theta^{0}∥}\right)\frac{Q_{ij}^{T}Q_{ij}\theta^{0}}{∥Q_{ij}\theta^{0}∥})-\sum_{j=1}^{M}2\frac{1}{\gamma_{j}^{2}}F_{j}^{T}\left({\tilde{s}}_{j}-F_{j}\theta \right)=0. \end{array}$$

Set

(A11a) $$\begin{array}{cc} \; & Q_{r}=\mathrm{diag}\left[\frac{1}{\sigma_{1,1}^{2}},\frac{1}{\sigma_{1,2}^{2}},\ldots ,\frac{1}{\sigma_{N,M}^{2}}\right],\;Q_{d}=\mathrm{diag}\left[\frac{1}{\beta_{1,1}^{2}},\frac{1}{\beta_{1,2}^{2}},\ldots ,\frac{1}{\beta_{N,M}^{2}}\right], \end{array}$$

(A11b) $$\begin{array}{cc} \; & Q_{s}=\mathrm{diag}\left[\frac{1}{\gamma_{1}^{2}},\frac{1}{\gamma_{2}^{2}},\ldots ,\frac{1}{\gamma_{M}^{2}}\right]⊗I_{p\times p},\;\tilde{s}={\left[{\tilde{s}}_{1}^{T},{\tilde{s}}_{2}^{T},\ldots ,{\tilde{s}}_{M}^{T}\right]}^{T},\;F={\left[F_{1}^{T},F_{2}^{T},F_{M}^{T}\right]}^{T}, \end{array}$$

(A11c) $$\begin{array}{cc} \; & h_{3}={\left[r_{11}-∥H_{1}\theta^{0}∥-∥P_{1}\theta^{0}∥,r_{12}-∥H_{1}\theta^{0}∥-∥P_{2}\theta^{0}∥,\ldots ,r_{NM}-∥H_{N}\theta^{0}∥-∥P_{M}\theta^{0}∥\right]}^{T}, \end{array}$$

(A11d) $$\begin{array}{cc} \; & h_{4}={\left[d_{11}-∥Q_{11}\theta^{0}∥,d_{12}-∥Q_{12}\theta^{0}∥,\ldots ,d_{NM}-∥Q_{NM}\theta^{0}∥\right]}^{T}, \end{array}$$

(A11e) $$\begin{array}{cc} \; & H_{3}=\left[\frac{H_{1}^{T}H_{1}\theta^{0}}{∥H_{1}\theta^{0}∥}+\frac{P_{1}^{T}P_{1}\theta^{0}}{∥P_{1}\theta^{0}∥},\frac{H_{1}^{T}H_{1}\theta^{0}}{∥H_{1}\theta^{0}∥}+\frac{P_{2}^{T}P_{2}\theta^{0}}{∥P_{2}\theta^{0}∥},\ldots ,\frac{H_{N}^{T}H_{N}\theta^{0}}{∥H_{N}\theta^{0}∥}+\frac{P_{M}^{T}P_{M}\theta^{0}}{∥P_{M}\theta^{0}∥}\right], \end{array}$$

(A11f) $$\begin{array}{cc} \; & H_{4}=\left[\frac{Q_{11}^{T}Q_{11}\theta^{0}}{∥Q_{11}\theta^{0}∥},\frac{Q_{12}^{T}Q_{12}\theta^{0}}{∥Q_{12}\theta^{0}∥},\ldots ,\frac{Q_{NM}^{T}Q_{NM}\theta^{0}}{∥Q_{NM}\theta^{0}∥}\right]. \end{array}$$

Then (A10) can be recast as

(A12) $$\begin{array}{c} H_{3}Q_{r}\left(h_{3}-H_{3}^{T}\left(\theta -\theta^{0}\right)\right)+H_{4}Q_{d}\left(h_{4}-H_{4}^{T}\left(\theta -\theta^{0}\right)\right)+F^{T}Q_{s}\left(\tilde{s}-F\theta \right)=0. \end{array}$$

Finally, the updating of θ is

(A13) $$\begin{array}{c} \theta ={\left(H_{3}Q_{r}H_{3}^{T}+H_{4}Q_{d}H_{4}^{T}+F^{T}Q_{s}F\right)}^{-1}\left(H_{3}Q_{r}\left(h_{3}+H_{3}^{T}\theta^{0}\right)+H_{4}Q_{d}\left(h_{4}+H_{4}^{T}\theta^{0}\right)+F^{T}Q_{s}\tilde{s}\right). \end{array}$$
